# MANAGEMENT OF SYMPTOMS RECURRENCE AFTER MYOTOMY FOR ACHALASIA. A PRACTICAL APPROACH

**DOI:** 10.1590/0102-672020230062e1780

**Published:** 2023-12-08

**Authors:** Francisco TUSTUMI, Sérgio SZACHNOWICZ, Nelson Adami ANDREOLLO, Francisco Carlos Bernal da Costa SEGURO, Edno Tales BIANCHI, André Fonseca DUARTE, Ary NASI, Rubens Antonio Aissar SALLUM

**Affiliations:** 1 Universidade de São Paulo, Department of Gastroenterology – São Paulo (SP), Brazil.; 2 Universidade Estadual de Campinas, Department of Surgery – Campinas (SP), Brazil.

**Keywords:** Esophageal Achalasia, Esophageal Diseases, Esophageal Motility Disorders, Reoperation, Dysphagia, Acalasia Esofágica, Doenças do Esôfago, Transtornos da Motilidade Esofágica, Reoperaçã, Disfagiao

## Abstract

**BACKGROUND:**

Achalasia is an esophageal motility disorder, and myotomy is one of the most used treatment techniques. However, symptom persistence or recurrence occurs in 9 to 20% of cases.

**AIMS:**

This study aims to provide a practical approach for managing the recurrence or persistence of achalasia symptoms after myotomy.

**METHODS:**

A critical review was performed to gather evidence for a rational approach for managing the recurrence or persistence of achalasia symptoms after myotomy.

**RESULTS:**

To properly manage an achalasia patient with significant symptoms after myotomy, such as dysphagia, regurgitation, thoracic pain, and weight loss, it is necessary to classify symptoms, stratify severity, perform appropriate tests, and define a treatment strategy. A systematic differential diagnosis workup is essential to cover the main etiologies of symptoms recurrence or persistence after myotomy. Upper digestive endoscopy and dynamic digital radiography are the main tests that can be applied for investigation. The treatment options include endoscopic dilation, peroral endoscopic myotomy, redo surgery, and esophagectomy, and the decision should be based on the patient’s individual characteristics.

**CONCLUSIONS:**

A good clinical evaluation and the use of proper tests jointly with a rational assessment, are essential for the management of symptoms recurrence or persistence after achalasia myotomy.

## RESUMO

**RACIONAL:**

A acalasia é um distúrbio da motilidade esofágica e a miotomia é uma das técnicas de tratamento mais utilizadas. No entanto, a persistência ou recorrência dos sintomas ocorre em 9 a 20%.

**OBJETIVOS:**

Este estudo visa fornecer uma abordagem prática para o manejo da recorrência ou persistência dos sintomas de acalasia após miotomia.

**MÉTODOS:**

Foi realizada uma revisão crítica para reunir evidências para uma abordagem racional no manejo da recorrência ou persistência dos sintomas de acalasia após miotomia.

**RESULTADOS:**

Para o manejo adequado de um paciente com acalásia com sintomas significativos após miotomia, como disfagia, regurgitação, dor torácica e perda de peso, é necessário classificar os sintomas, estratificar a gravidade, realizar exames adequados e definir uma estratégia de tratamento. Uma investigação diagnóstica diferencial sistemática é essencial para cobrir as principais etiologias de recorrência ou persistência dos sintomas após a miotomia. A endoscopia digestiva alta e a radiografia digital dinâmica são os principais exames que podem ser aplicados para investigação. As opções de tratamento incluem dilatação endoscópica, POEM (miotomia endoscópica oral), remiotomia e esofagectomia, e a decisão deve ser baseada nas características individuais do paciente.

**CONCLUSÕES:**

Uma boa avaliação clínica e a utilização de exames adequados, juntamente com uma avaliação racional, são essenciais para o manejo da recorrência ou persistência dos sintomas após miotomia por acalasia.

## INTRODUCTION

A chalasia is characterized by the dysfunction of esophageal motility and lower esophageal sphincter (LES) functioning, which fails to relax properly during swallowing, leading to impaired passage of food and liquids into the stomach^3^. The result is a myriad of distressing symptoms, including dysphagia, regurgitation, chest pain, and weight loss. 

The etiology of achalasia remains an intriguing puzzle in the realm of Gastroenterology. While the precise cause of this complex esophageal disorder is not fully understood, it is widely believed to be multifactorial, involving genetic and environmental factors^30^. Infections, such as herpes simplex virus and *Trypanosoma cruzi*, have been implicated, potentially triggering an immune response leading to the degeneration of the ganglion cells in the myenteric plexus, a key component of esophageal motility^30^. Furthermore, genetic predisposition has been observed, with familial clustering and the identification of certain genetic markers associated with an increased risk of developing the condition^8^. While the precise etiology of achalasia continues to elude us, ongoing research seeks to unravel the intricate interplay of these factors, offering hope for improved understanding and more targeted approaches to its management.

Treating achalasia involves a multifaceted approach that aims to alleviate symptoms and enhance the patient’s overall quality of life. Endoscopic dilation, peroral endoscopic myotomy (POEM), and laparoscopic myotomy are the most traditional treatments for achalasia. Other non-conventional treatment alternatives, including botulinum toxin injections and cardioplasty, are supported only by a few pieces of evidence^26,31^. The choice of care modality depends on various factors, including the patient’s overall health, the severity of symptoms, and the expertise of the treating physician. 

Myotomy has proven to be a safe and highly effective care option for achalasia. This surgical intervention involves carefully cutting the LES muscle to alleviate the obstructive symptoms. Myotomy can significantly improve the quality of life for achalasia patients by relieving dysphagia, regurgitation, and chest pain. The predicted probability for improvement in dysphagia at 12 months is 91%, and at 24 months, 90%^11,20^. 

However, symptom persistence or recurrence after myotomy is not uncommon and occurs in 9 to 20% of cases^2,23^. The reasons for recurrence are vast, and the risk of treatment failure depends on the preoperative achalasia grade. An advanced megaesophagus has a 12.8% retreatment risk after Heller myotomy^16^. The monitoring of patients with symptoms persisting or recurring after a surgical myotomy may be challenging. Manuscripts that orientate management standardization are essential to help gastroenterologists, surgeons, and endoscopists properly diagnose and treat factors associated with symptoms persistence or recurrence, such as incomplete myotomy, valve migration, end-stage achalasia, cancer, and others.

This manuscript presents our view and perspective on managing symptom persistence or recurrence after myotomy for achalasia. 

## METHODS

This critical review gathers scientific evidence to build a rational approach for managing the recurrence or persistence of achalasia symptoms after myotomy. The literature search and selection were performed in PubMed, Embase, and LILACS databases to construct this review, and a critical view from the authors was considered.

### Management of achalasia symptoms recurrence or persistence after myotomy

There is no definitive strategy for patient monitoring and not even an optimal time for repeated operations for patients with achalasia symptoms recurrence or persistence after myotomy. Our manuscript presented our experienced view and rationality for managing achalasia symptoms recurrence after myotomy. 

Firstly, a thorough clinical history should be obtained before making any decision. The indications for a new intervention for achalasia should be guided mainly by patients’ symptoms and quality of life impairment. Consequently, it is imperative that any decision concerning new procedures should be grounded on the principle of shared decision-making. Healthcare professionals should use their experience and knowledge directed at the unique perspectives and values of the patient. Shared decision-making ensures that individuals are not passive recipients of medical interventions but active participants in their healthcare journey. By engaging in open and honest discussions, patients can gain a comprehensive understanding of the potential risks, benefits, and alternatives associated with surgical procedures. In turn, this empowers them to make informed choices that align with their preferences and goals, fostering a sense of agency and trust in the healthcare system. Ultimately, shared decision-making promotes patient-centered care, enhancing the overall quality and satisfaction of healthcare outcomes^10^. 

Considering that achalasia treatment is a palliative intervention and esophageal function does not normalize after treatment, patients are expected to have mild symptoms even after a well-performed myotomy. However, significant symptoms or deterioration of quality of life may point to a disturbance that may demand retreatment. The main symptoms are dysphagia, regurgitation, and thoracic pain, and each of these symptoms may indicate a different issue. 

After a comprehensive clinical history, proper tests are essential to establish a rational strategy for patient care. Inappropriate anamnesis may induce misdiagnosis. Several patients with achalasia symptoms are mistreated as gastroesophageal reflux disease or psychiatric disorders.

For proper management of an achalasia patient with symptoms after myotomy, we suggest the following evaluation steps:

### Classify the symptoms

**Figure 1:**
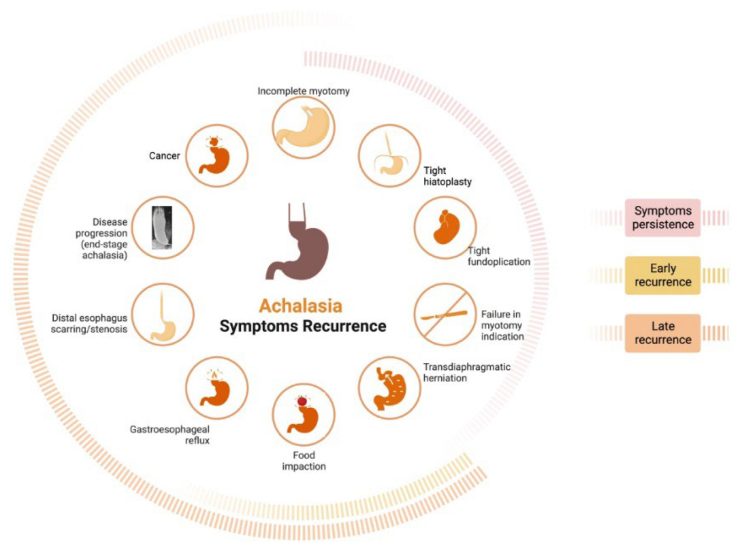
Symptoms after myotomy should be classified into one of three categories: “symptoms persistence”, “early recurrence”, or “late recurrence”. These categories help healthcare professionals to work up differential diagnoses.

We proposed a rationality for managing symptoms persistence or recurrence in achalasia. Basically, we classify symptoms into three categories: “symptoms persistence”, “symptoms early recurrence”, and “symptoms late recurrence”^15^. These categories help healthcare professionals to work up differential diagnoses (Figure 1, Table 1).

**Table 1:** Classification of symptoms after myotomy.

Temporality	Differential diagnosis	Management*
Dysphagia persistence	Incomplete myotomy Tight hiatoplasty Tight fundoplication Failure in myotomy indication	Endoscopic dilation, redo surgery, POEM, esophagectomy Redo surgery Redo surgery Esophagectomy
Dysphagia early recurrence	Transdiaphragmatic herniation Food impaction	Redo surgery Endoscopy
Late recurrence	Transdiaphragmatic herniation Food impaction Distal esophagus scarring Esophageal stenosis Gastroesophageal reflux Disease progression (end-stage achalasia) Cancer	Redo surgery Endoscopy Endoscopic dilation Endoscopic dilation Medical therapy, redo surgery Esophagectomy Tailored decision

* The choice for the treatment strategy should be based on a good clinical evaluation and proper tests, jointly with a rational assessment. POEM: peroral endoscopic myotomy.

The cause of the symptoms’ persistence usually is failed surgery. The main reason for failed surgery is an incomplete myotomy. Other possible diagnoses include thigh fundoplication or thigh hiatoplasty (leading to obstruction). Another possibility is the inappropriate myotomy indication. In this situation, the patient has end-stage achalasia and will probably demand an esophagectomy.

Early recurrence may be caused by valve migration through hiatus. Usually, the symptoms are acute, with intense retrosternal pain. Food impaction may also occur. It is common for achalasia patients to change their oral diet drastically after a myotomy and start to ingest solid foods that may be difficult to pass through the cardia, even after a well-performed myotomy.

For late recurrence, probably the major concern is the development of esophageal cancer. Differential diagnoses comprise food impaction, end-stage achalasia, and peri-esophageal sclerosis^15^. Patients with intense regurgitation after myotomy may be related to gastroesophageal reflux, which is more common in patients with no anti-reflux valve or with a disrupted valve^2,18^. 

### Stratify symptoms severity

Before embarking on a treatment plan for achalasia, a critical and essential step in the decision-making process is the thorough assessment of the disease’s grade and severity. Achalasia can manifest in varying degrees of functional impairment, and its treatment approach should be tailored to each individual’s specific condition and impact on quality of life. By meticulously assessing the achalasia grade, healthcare providers can make informed decisions, optimize treatment strategies, and improve the overall outcomes and quality of life for individuals afflicted with this challenging esophageal disorder. 

The Eckardt is the most used score to stratify symptom severity (Table 2)^7^. It is based on weight loss, chest pain, regurgitation, and dysphagia^7,21^. Each item is graded 0–3. The Eckardt score helps to evaluate the efficacy of the achalasia treatment. An Eckardt score >3 after myotomy suggests surgery failure and usually demands retreatment^21,24^. 

**Table 2:** The Eckardt symptom score^7^.

Eckardt symptom score				
Score	Dysphagia	Regurgitation	Retrosternal pain	Weight loss (kg)
0	None	None	None	None
1	Occasional	Occasional	Occasional	<5
2	Daily	Daily	Daily	5–10
3	Each meal	Each meal	Each meal	>10

Currently, assessing achalasia outcomes relies heavily on the Eckardt score. Nevertheless, the Eckardt score has limitations. For example, in this score, weight loss is not based on a percentage change from the baseline patients’ weight. Besides, patients often undergo various changes following myotomy, including valve disruption, the development of gastroesophageal reflux disease (GERD), strictures, and other complications that may induce symptoms that are not comprised within the Eckardt score. Consequently, the Eckardt score should be complemented by other clinical evaluations, including investigating the impact of achalasia symptoms on quality of life. Other scores are reported in the literature to evaluate the various etiologies of dysphagia^19^.

Urbach et al. developed the achalasia disease-specific health-related quality of life (HRQoL) questionnaire, which consists of ten items that assess various aspects of a patient’s well-being (Table 3)^27^. These items encompass food tolerance (items 1 and 2), dysphagia-related behavioral modifications (item 5), pain (item 6), heartburn (item 7), distress (item 8), lifestyle limitations (item 9), and satisfaction (item 10). The scoring system assigns different point values to each item, reflecting the severity or impact of the symptom. The total score can range from a minimum of 10 points to a maximum of 31, with a lower score indicating a better disease-specific HRQoL. This point system can be adjusted to a scale of 0–100, if necessary. The HRQoL questionnaire was validated in other studies^5,18,22^.

**Table 3:** Achalasia disease-specific health-related quality of life (HRQoL) questionnaire^27^.

1. How much has achalasia limited the types of food you have been able to eat in the last month? a. Not limited at all (I can eat and drink all the foods that I would like to) b. Somewhat limited (I can eat and drink most of the foods that I would like to) c. Moderately/Severely limited (I can eat and drink very few of the foods I would like to)
The following is a list of food types that may or may not cause you difficulty in swallowing. Please indicate which of the following types of foods you are able to swallow without experiencing any problems such as pain or food ‘sticking’ as it goes down. If you are not sure whether you can swallow a type of food without problems, please make you best guess.
2. Raw hard fruits and vegetables a. Can swallow without problem b. Can swallow, but with some difficulty c. Can swallow with great difficulty or not at all
3. Rice a. Can swallow without problem. b. Can swallow, but with some difficulty c. Can swallow with great difficulty or not at all
4. Clear fluids (water, juice, coffee, tea) a. Can swallow without problem. b. Can swallow, but with some difficulty c. Can swallow with great difficulty or not at all
5. How often in the past month have you need to drink water while eating to deal with food caught in your esophagus? a. Never/Rarely b. Sometimes c. Frequently/every time I eat
6. How often have you experienced pain when eating during the past month? a. Never b. Rarely c. Sometimes d. Frequently/every time I eat
7. During the past month, how much of a problem was heartburn (a burning pain behind the lower part of the chest) for you? a. No problem b. Mild problem c. Moderate problem d. Severe problem e. Very severe problem
8. When you sit down to eat a meal, are you bothered by how long it takes you to finish eating? a. No, I eat as quickly as I like b. Yes, I am bothered by how long it takes me to eat
9. Has having achalasia limited your lifestyle? a. No, it is not at all limiting (My daily activities have not changed) b. Yes, it has limited my lifestyle (it has affected some areas, and I can no longer participate in all activities I want to do)
10. How much do you agree with the following statement about how satisfied you are with your health in regard to achalasia? I am satisfied with my health in regard to achalasia. a. Strongly agree b. Agree c. Neither agree or disagree d. Disagree e. Strongly disagree

However, achalasia patients are complex and may manifest a myriad of heterogenic symptoms. Ergo, the clinical evaluation should be individualized, and a combination of scores and questionnaires may be necessary. Other commonly used scores for evaluating post-myotomy quality of life are the Dakkak Dysphagia score and the GERD-HRQL score to quantify dysphagia and GERD, respectively^21^.

### Perform proper tests

Firstly, if available, the video of the primary surgery should be watched, looking for technical surgical mistakes, such as short myotomy, thigh fundoplication or hiatoplasty.

**Figure 2:**
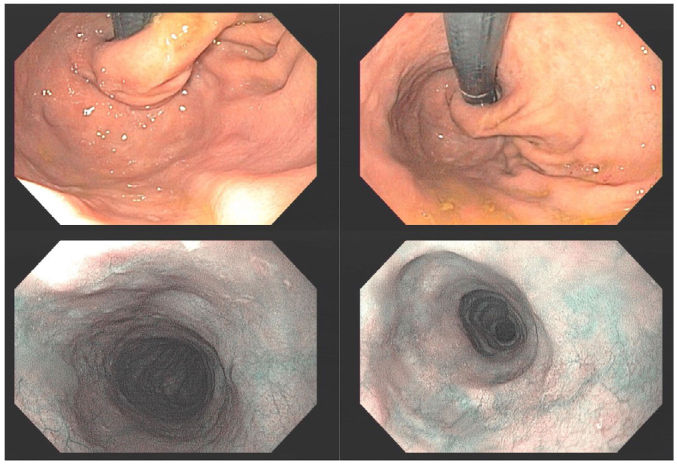
Endoscopy from a patient with achalasia after a myotomy with partial fundoplication. The esophagus presents a body with augmented diameter and tortuosity, with no malignancy suspicion at the chromoendoscopy. The retroflexed view shows a normal partial fundoplication. The patient was satisfactorily treated with peroral endoscopic myotomy. The anti-reflux valve hampered the risk of post-peroral endoscopic myotomy gastroesophageal reflux.

**Figure 3:**
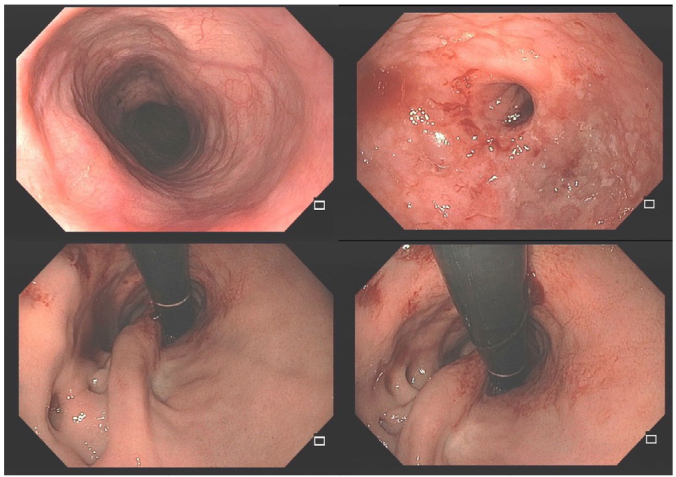
Achalasia patient with disruption of the anti-reflux valve and distal erosive esophagitis. This patient had ineffective endoscopic dilation treatments. After a redo laparoscopic surgery, with a new myotomy and a new partial fundoplication, the patient presented improvement in dysphagia and gastroesophageal reflux.

The patient should be assessed with an endoscopy and a barium swallow test. Endoscopy shows if there are fundoplication disruptions and investigates for neoplasms. Besides, upper endoscopic evaluations may detect the presence of esophagitis as graded by the Los Angeles Classification system. Bianchi et al.^2^ found that fundoplication disruption is closely related to esophagitis, which may explain certain symptoms recurrence (Figures 2 and 3). 

**Figure 4:**
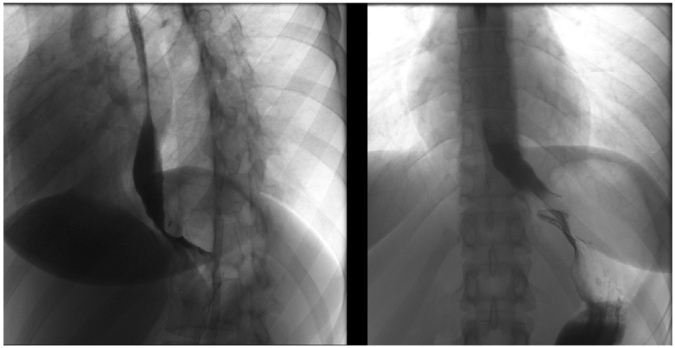
In this patient with incomplete myotomy, the barium swallow test showed no sign of herniation and a normal fundoplication. This patient failed treatment with balloon dilation and was finally treated with laparoscopic myotomy and partial fundoplication. Peroral endoscopic myotomy could be a reasonable alternative.

**Figure 5:**
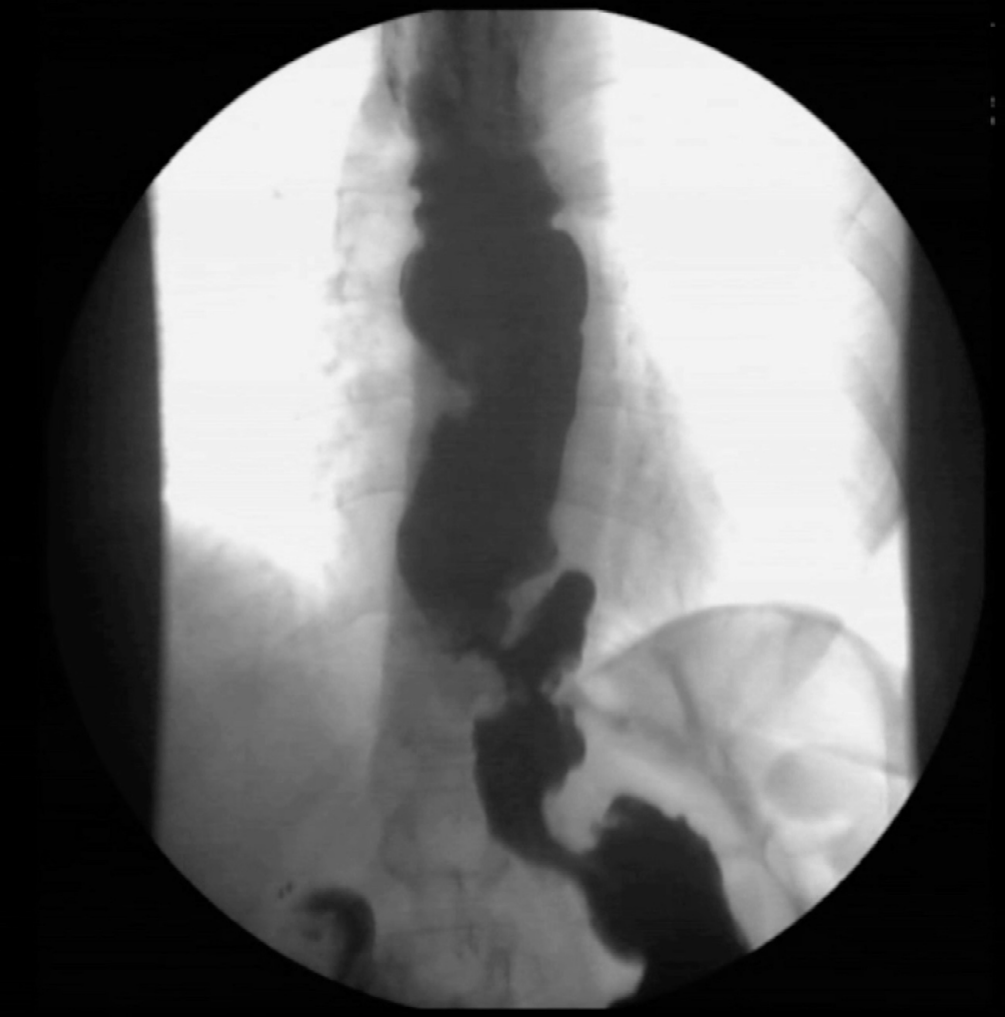
The barium swallow test shows an esophagus with augmented diameter and tortuosity. The imaging test suggests that small herniation through hiatus influenced dysphagia recurrence after myotomy. A peroral endoscopic myotomy would not solve the herniation problem, and consequently, the best approach was laparoscopic revisional surgery. The hernia was corrected, and a new myotomy was performed.

**Figure 6:**
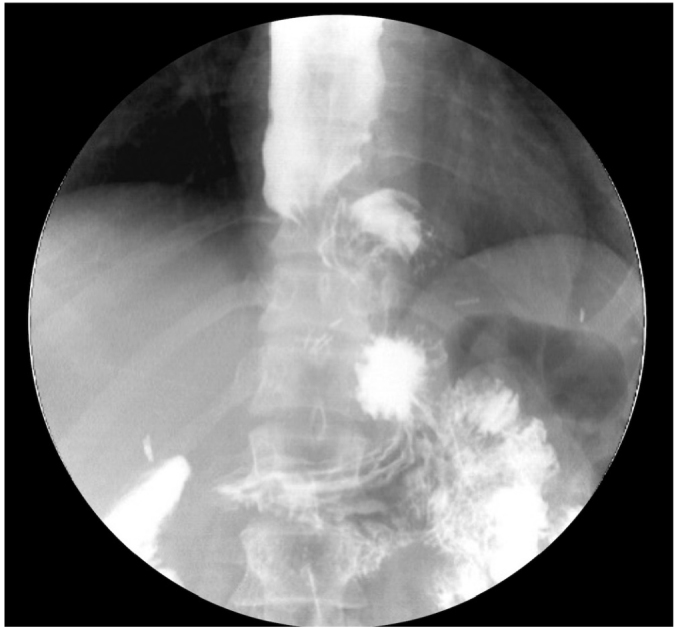
This contrast radiography shows a significant valve migration. Endoscopic procedures would not correct the valve migration, and ergo, were not considered. The patient was treated with a revisional laparoscopic surgery.

A barium swallow test helps detect if a hiatal hernia contributes to dysphagia. A dynamic digital radiography is better than a static test since it allows the evaluation of esophageal clearance. The clearance time of barium over 5 minutes indicates an inefficient alimentary bolus transport^21^. Endoscopy and contrast radiography are the most critical tests for the evaluation of symptoms after myotomy (Figures 4, 5, and 6). 

High-resolution manometry post-myotomy may be evaluated. The mean integrated relaxation pressure (IRP) >15 mmHg indicates that maybe a previous myotomy was not efficient, and the patient probably needs a redo myotomy. However, if a fundoplication was associated with the primary myotomy surgery, the IRP value may be influenced by the anti-reflux valve functioning^12,18^. 

A pH-meter test with the patient off anti-secretory drugs or 24-hour impedance may be considered only in selected patients^21^. A composite DeMeester score >14.72 indicates esophageal acid reflux exposure. However, pH tests are complex to interpret in achalasia disease since fermentation due to esophageal stasis induces a pH drop, creating a significant confounding variable^14^. 

### Define the approach

Patti et al. recommend that endoscopic dilation should be the first-line treatment for symptom recurrence or persistence after myotomy for achalasia. However, we ponder that the decision should be based on a wide range of considerations since endoscopic dilation will fatefully fail in some situations, such as sigmoid-shaped achalasia, hiatal herniation, and obstructing fundoplication^17,18^. The treatment should be based on thorough clinical and diagnostic tests findings. 

Symptom persistence usually represents a surgical technical failure. If patient has sigmoid-shaped achalasia and fails after myotomy, probably the patient will demand an esophagectomy or an alternate surgical method for end-stage achalasia, such as Serra-Dória or Thal-Hatafuku^26^. These procedures are high-risk. Esophagectomy for achalasia is associated with a risk of 2–5% mortality and 30% morbidity. Consequently, choosing any of these alternatives should only be considered in end-stage achalasia when previous treatment alternatives fail. These procedures should be performed only by experienced surgical centers for improved probability of success^13,26,28^.

In the case of an incomplete myotomy, the patient will demand a reintervention. Endoscopic dilation can be used as a therapeutic test. A parcel of patients will show symptom improvement after 1–4 dilations, but some will have recurrences or show no significant improvement^32^. In these latter patients, a redo myotomy will be necessary^32^ It can be performed either by endoscopy (POEM), with less morbidity, or by a transabdominal approach (laparoscopic or robotic-assisted)^9^. Transabdominal will be the approach of choice if preoperative tests show an anatomical change, such as a large hiatal hernia. 

If the previous myotomy was associated with fundoplication, POEM is an exciting option since it avoids the esophagogastric adhesions of transabdominal access. If a patient has a previous fundoplication, the POEM risk for reflux will be mitigated^9^. In these cases, it is essential that upper endoscopy shows a fair fundoplication with no signs of disruption. 

Conversely, transabdominal access gives a global picture of the previous failed surgery. A laparoscopy can confirm whether a thigh hiatoplasty or fundoplication contributed to the persistence of symptoms. Unlike POEM, transabdominal access may correct valve migration (Figures 5 and 6).

As a limitation, if the redo myotomy fails again, an eventual new surgery for end-stage achalasia will be much more demanding. If the patient is referred for an esophagectomy, the hiatus might present dense fibrosis tissues. Besides, an eventual gastric pull-up technique will be challenging due to the fundoplication.

Currently, there is no consensus for POEM or laparoscopic redo myotomy in patients with symptoms recurrence or persistence. Akimoto et al. compared POEM to redo myotomy after a failed myotomy^1^. The authors concluded that POEM and laparoscopic redo surgery were equivalent for postoperative satisfaction and dysphagia control, but POEM had lower short-term complications, while laparoscopic redo surgery had less esophagitis. However, the sample size was significantly small, and the studied groups were exceedingly heterogeneous. Only future randomized trials will determine what is the best option for a redo surgery for a failed myotomy, whether POEM or laparoscopic myotomy.

Milito et al. described their 20-year experience with revisional surgery for achalasia, with one cardioplasty, two esophagectomies, and ten redo laparoscopic myotomies^13^. Despite significant improvement after revisional surgery, 23% of patients complained of occasional dysphagia at long-term follow-up. Costa et al. presented their experience with 26 achalasia patients with symptoms recurrence. Most patients were treated with a redo myotomy (53%) or a Serra-Dória procedure (30.7%). The authors stated that 80% of patients showed significant symptom improvement after revisional surgery^4^. 

If a transabdominal myotomy is decided, some technical details during surgery should be followed. Surgeons should investigate for any signs of esophagogastric obstruction, such as thigh fundoplication or hiatoplasty, and then undo the previous fundoplication, providing complete exposure of the esophageal wal, redo myotomy with at least 7 cm length, extending 2–3 cm below the gastroesophageal junction, ensuring that the submucosa is well exposed, with cardia muscle fibers well apart. The surgeon can decide on a new myotomy in a different site or an extension of the previous one. Avoiding the previous myotomy may ease the dissection by escaping the adhesions generated by the previous scar. Finally, the surgeon ideally should create a partial valve covering the myotomy, carefully avoiding obstruction in the new fundoplication, although there has yet to be a consensus on the type of valve among specialists. Despite no existing controlled studies, robotic platforms could be helpful for revisional surgery if available due to the enhanced visualization and movement degrees of freedom^6^. 

Thoracoscopic myotomy via the left chest is another strategy that can be used to avoid abdominal adhesions^29^. If the first myotomy was performed with a POEM procedure, abdominal adhesions would not be an issue, and redo transabdominal surgery would be the treatment of choice. 

Soluble contrast radiography should be performed for patients with early recurrence within days or weeks after surgery. Acute trans-hiatal valve migration can be a life-threatening condition and demands early intervention. Usually, patients with acute valve migration present with worsening dysphagia and acute thoracic pain, which is more common after intense abdominal strain exercise.

Food impaction is also common after a myotomy. Usually, patients present with dysphagia worsening, but with no pain. It is common for achalasia patients to change their oral diet drastically after a myotomy and start to ingest solid foods that may be difficult to pass through the cardia. These patients should be evaluated with an upper endoscopy, and if food impaction is diagnosed, the endoscopic food removal should solve the problem. Therefore, it is reasonable to orient patients to follow a careful diet in the first days after myotomy, with liquid meals, chewing the food properly, and avoiding copious meals. 

Significant regurgitation after long-term follow-up associated with esophagitis signs at endoscopy is usually related to anti-reflux valve disruption^2^. Endoscopic dilation or POEM can worsen this symptom, and the best treatment is a redo surgery with a new partial fundoplication.

Dysphagia recurrence after long-term follow-up after a myotomy should always raise a red flag for cancer. Achalasia is a known risk factor for cancer, even after myotomy^25^. The patient should undergo an upper endoscopy with a chromoendoscopy and biopsy. If cancer is diagnosed, proper staging tests should be performed. The management should be based on an individualized approach in a multidisciplinary oncologic meeting. 

Finally, it is important to advise patients that achalasia is an incurable condition, and treatment will only improve symptoms and not improve their esophageal function. Mild symptoms are common and acceptable after achalasia treatment, and few patients report no symptoms. Despite several authors stating that an Eckardt score ≤4 implies satisfactory symptoms control, the assessment of treatment success should not be based on a single score, instead, it should be found on the entire clinical evaluation and quality of life assessment.

## DISCUSSION

This manuscript presented our experience in the management of symptoms recurrence after myotomy. Currently, there is scarce evidence in the literature regarding revisional procedures for achalasia. Most studies are non-analytical and provide unclear treatment algorithms.

Due to the small sample sizes in published studies, it is hard to determine all the driving factors associated with better or worse outcomes. Knowing these variables would help select candidates for redo surgery and predict outcomes. Probably, many variables, such as age, sex, achalasia etiology, or manometric subtypes may influence outcomes after redo surgery and, theoretically, could partially explain why some patients have poor symptom control. Future multicenter studies are necessary to identify these variables and help select patients for any revisional procedure for achalasia.

## CONCLUSIONS

A good clinical evaluation and proper tests, jointly with a rational assessment, are essential for managing symptoms recurrence or persistence after achalasia myotomy.
